# The Ganglioside Monosialotetrahexosylganglioside Protects Auditory Hair Cells Against Neomycin-Induced Cytotoxicity Through Mitochondrial Antioxidation: An *in vitro* Study

**DOI:** 10.3389/fncel.2021.751867

**Published:** 2021-09-27

**Authors:** Yujin Li, Ao Li, Chao Wang, Xin Jin, Yaoting Zhang, Ling Lu, Shou-Lin Wang, Xia Gao

**Affiliations:** ^1^Department of Otolaryngology-Head and Neck Surgery, Nanjing Drum Tower Clinical College of Nanjing Medical University, Nanjing, China; ^2^Department of Otolaryngology-Head and Neck Surgery, The Affiliated Huaian No. 1 People’s Hospital of Nanjing Medical University, Huai’an, China; ^3^Research Institute of Otolaryngology, Nanjing, China; ^4^Jiangsu Provincial Key Medical Discipline (Laboratory), Department of Otolaryngology-Head and Neck Surgery, Affiliated Drum Tower Hospital of Nanjing University Medical School, Nanjing, China; ^5^School of Public Health, Nanjing Medical University, Nanjing, China

**Keywords:** monosialotetrahexosylganglioside, neomycin, hair cell, cytotoxicity, reactive oxygen species

## Abstract

Neomycin is a common ototoxic aminoglycoside antibiotic that causes sensory hearing disorders worldwide, and monosialotetrahexosylganglioside (GM1) is reported to have antioxidant effects that protect various cells. However, little is known about the effect of GM1 on neomycin-induced hair cell (HC) ototoxic damage and related mechanism. In this study, cochlear HC-like HEI-OC-1 cells along with whole-organ explant cultures were used to establish an *in vitro* neomycin-induced HC damage model, and then the apoptosis rate, the balance of oxidative and antioxidant gene expression, reactive oxygen species (ROS) levels and mitochondrial membrane potential (MMP) were measured. GM1 could maintain the balance of oxidative and antioxidant gene expression, inhibit the accumulation of ROS and proapoptotic gene expression, promoted antioxidant gene expression, and reduce apoptosis after neomycin exposure in HEI-OC-1 cells and cultured cochlear HCs. These results suggested that GM1 could reduce ROS aggregation, maintain mitochondrial function, and improve HC viability in the presence of neomycin, possibly through mitochondrial antioxidation. Hence, GM1 may have potential clinical value in protecting against aminoglycoside-induced HC injury.

## Introduction

Sensorineural hearing loss is a common sensory disorder in humans. Many factors, including viral infections, noise, and exposure to ototoxic drugs, can cause hair cell (HC) damage and induce hearing impairment (Sotoudeh, 2021). Adult mammalian HCs cannot regenerate, and once these cells are damaged, the effects are often permanent (Zhang et al., 2020). Aminoglycoside antibiotics are antibacterial drugs commonly used in clinical treatment and are also the most commonly encountered ototoxic drugs. It is estimated that approximately one-quarter of people who are treated with aminoglycoside antibiotics will develop ototoxicity (Lopez-Novoa et al., 2011). However, these drugs still play a crucial role in the treatment of severe gram-negative bacterial infections and multidrug-resistant tuberculosis and are becoming more irreplaceable as microbial resistance to conventional antimicrobial agents increases (Germovsek et al., 2017). Therefore, exploring the mechanism through which they exert ototoxicity and identifying effective treatment measures is of great significance (Guo et al., 2019). The ototoxic mechanism of aminoglycoside drugs has not been fully elucidated. Recent studies have found that it may be related to oxidative stress (Jiang et al., 2016), apoptosis (Jiang et al., 2017), and mitochondrial dysfunction (Jiang et al., 2017). Previous studies have found that aminoglycoside drugs accumulate in mitochondria after entering HCs and that this can lead to disorders of mitochondrial metabolism, imbalanced expression of prooxidants (ALOX15) and antioxidants (GSR, SOD1, NQO1, GLRX), and the production of excessive reactive oxygen species (ROS) such as hydrogen peroxide (H_2_O_2_) and hydroxyl radicals (∙OH) in cells. Aminoglycosides also disrupt the normal synthesis of mitochondrial proteins and reduce the mitochondrial membrane potential (MMP), leading to increased mitochondrial permeability and release of cytochrome C and ultimately to cell apoptosis (He et al., 2020; Yao et al., 2020). It has been reported that some antioxidants exert protective effects against the ototoxicity of aminoglycosides *in vitro* and *in vivo* (Li et al., 2018; Pham et al., 2020). However, no ideal drug that is protective against ototoxicity has been identified for use in clinical practice (Guo et al., 2019).

Gangliosides are sphingolipid cell membrane components containing sialic acid (Magistretti et al., 2019) that are widely present in the cell membranes of vertebrate tissues. Monosialotetrahexosylganglioside (GM1) is the most extensively studied of the gangliosides to date. GM1 plays a protective role in the nervous system by regulating the expression of BCL-2 protein, reducing the damage caused by free radicals (Rao et al., 2019), increasing mitochondrial activity and stabilizing mitochondrial function (Fazzari et al., 2020). GM1 has also been found to have a cytoprotective effect outside of the nervous system. Franco et al. (2016) found that GM1-containing nanoliposomes protect against light chain protein (LC)-induced human microvascular dysfunction by increasing nitric oxide bioavailability and reducing oxidative and nitrative stress mediated by the Nrf-2-dependent antioxidant stress response. Guo et al. (2021) reported that treatment with GM1 ganglioside (40 μM) significantly decreased α-synuclein accumulation, alleviated mitochondrial dysfunction and oxidative stress, and played an anti-PD role in doxycycline-treated PC12α-Syn A53T cells. In addition, GM-1 treatment was found to significantly decrease auditory brainstem response (ABR) threshold shifts and HC loss after acoustic overexposure, and immunostaining for 4-hydroxynonenal (4-HNE) was found to be reduced by GM-1 treatment, suggesting that GM-1 protects the cochlea against acoustic injury by inhibiting lipid peroxidation (Tanaka et al., 2010) and indicating that GM1 has an antioxidant effect on cochlear HCs.

GM1 has been used as a neuroprotective drug in some countries for the treatment of Alzheimer’s disease, stroke, peripheral nerve damage, and Parkinson’s disease (Magistretti et al., 2019), and some physicians in China are attempting to use GM1 in the clinical treatment of sudden deafness (Diao and Dong, 2012). However, the protective effect of GM1 against cochlear HC damage and the possible mechanism of this effect are still unclear. Therefore, clarification of the protective effect and mechanism of GM1 on cochlear and HC injury is of practical significance.

Neomycin is a representative ototoxic aminoglycoside drug and is often used in ototoxicity studies. We established a mature neomycin HC injury model in previous experiments (Gao et al., 2019). In the present study, we used neomycin to damage cochlear HC-like HEI-OC-1 cells and used whole-organ explant cultures to establish an *in vitro* model of neomycin-induced HC damage with the aim of investigating the potential protective effect of GM1 on ototoxic HC damage and attempting to provide evidence for the potential clinical use of GM1 in protecting against HC injury caused by aminoglycosides.

## Materials and Methods

### Chemicals and Reagents

GM1 (Macklin, Shanghai, China, G873919), penicillin (CSPC, Shijiazhuang, China, h20033291), CCK-8 Kits (Beyotime, Shanghai, China, C0038), BeyoRT^TM^ II First-Strand cDNA Synthesis Kits with gDNA Eraser (Beyotime, D7170 M), TUNEL Apoptosis Assay Kits (Beyotime, C1086), DMSO (Sigma-Aldrich, Saint Louis, MO, United States, D8371), neomycin (Sigma-Aldrich, N6386-5G), paraformaldehyde (Sigma-Aldrich, 158127), TRIzol reagent (Sangon Biotech, Shanghai, China, B610409-0100), SYBR Green (Roche, Basel, Switzerland, 4913914001), MitoSOX Red (Life Technologies, Carlsbad, CA, United States, M36008), TMRE Mitochondrial Membrane Potential Assay Kits (Abcam, Cambridge, United Kingdom, ab113852), Triton X-100 (Solarbio, Beijing, China, T8260), DAPI (Solarbio, C0060), antibodies against cleaved CASPASE-3 (Thermo Fisher Scientific, Carlsbad, CA, United States, Cat# 43-7800, RRID:AB_2533540), antibody against MYOSIN 7a (Santa Cruz Biotechnology, Santa Cruz, United States, Cat# sc-74516, RRID:AB_2148626), Alexa Fluor 488 goat anti-rabbit IgG (Abcam, Cat# ab150077, RRID:AB_2630356), Alexa Fluor 555 donkey anti-rabbit IgG (Abcam, Cat# ab150062, RRID:AB_2801638), Alexa Fluor 555 goat anti-mouse IgG (Abcam, Cat# ab150118, RRID:AB_2714033), and Alexa Fluor 647 donkey anti-rabbit IgG (Abcam, Cat# ab150077, RRID:AB_2630356) were used in this study.

### Cell Culture and Treatment

**FIGURE 1:**
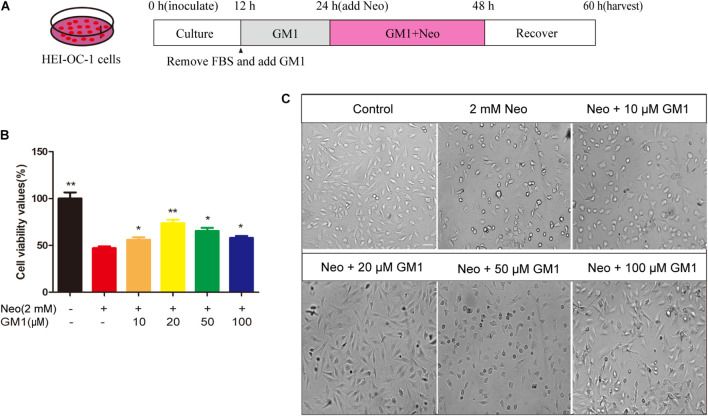
GM1 enhanced HEI-OC-1 cell survival after neomycin exposure. **(A)** Schematic diagram illustrating GM1 and neomycin addition in cell culture. **(B)** Effects of GM1 on the viability of HEI-CO-1 cells after exposure to neomycin. Viability was measured using the CCK-8 assay, and the data are expressed as the mean ± SD of triplicate determinations obtained in three independent experiments. *p < 0.05, **p < 0.01 compared with neomycin treatment alone. **(C)** Morphology of HC-like HEI-OC-1 cells observed under a live-cell imaging system. Scale bars = 20 μm.

The House Ear Institute Organ of Corti 1 cell line (HEI-OC-1, RRID: CVCL_D899), which is used as an HC-like model system, expresses Atoh1, Prestin, Myosin7a and other cellular markers specific for auditory and sensory HCs. The cells were cultured in DMEM/F12 containing 10% fetal bovine serum (FBS) and 100 IU/mL penicillin at 37°C in a 5% CO_2_ atmosphere. In this study, we exposed cells in FBS-free culture medium to 2 mM neomycin for 24 h to establish an HEI-OC-1 cell injury model, as reported previously (Gao et al., 2019). For the establishment of the HEI-OC-1 cell injury protection model, we plated the cells, cultured them for 12 h, replaced the medium with FBS-free culture medium, and treated the cells with 10–100 μM GM1 or with the corresponding volume of DMSO for 12 h. After 24 h treatment with 2 mM neomycin, the medium was replaced with complete culture medium lacking neomycin and GM1, and the cells were cultured for an additional 12 h before subsequent evaluation (Figure 1A).

### Whole-Organ Explant Culture

On postnatal day 3 (P3), wild-type FVB mice were placed under deep anesthesia and sacrificed, and the animals’ heads were removed and placed in 75% alcohol. The temporal bone cochlea was removed using scissors and placed in a sterile culture dish containing precooled HBSS buffer. Under microscopic view, the volute covering was peeled off, and the spiral ganglia and vascular stripes were separated to obtain a complete basilar membrane, which was then placed face up in the center of a sterile cover glass coated with Cell-tak tissue glue. The cover glass was placed in a 4-well plate and cultured in 120 μL of DMEM/F12 medium containing 2% B27, 1% N-2 and 100 IU/mL penicillin at 37°C in a 5% CO_2_ atmosphere. The cochlear HC damage condition was 0.5 mM neomycin for 12 h, as reported previously (Gao et al., 2019). The explants in the GM1 group were cultured in medium to which 20 μM GM1 had been added; after 12 h, 0.5 mM neomycin was added, and the explant was cultured for an additional 12 h. The medium containing GM1 and neomycin was then removed, and the explants were cultured in fresh medium for an additional 12 h for recovery. The control group and the neomycin group were treated with the same volume of DMSO. All procedures involving FVB mice were performed in accordance with the procedures approved by the Animal Care and Use Committee of Nanjing Medical University, and all efforts were made to use the minimum number of animals necessary and to minimize their suffering.

### Cell Viability and Number Analysis

Cell viability was measured using a CCK-8 kit. HEI-OC-1 cells were seeded in 96-well plates at 2,000 cells/well. After the treatment described above, 10 μL of CCK-8 was added to each well, and the plates were incubated at 37°C for 30 min. The absorbance at 450 nm was measured using a microtiter plate reader (Bio-Rad). In addition, the HCs of explant-cultured cochleae were immunolabeled using antibodies against MYOSIN 7a and counted by fluorescence labeling microscopy. The MYOSIN 7a-positive cells in three regions of equal length (160 μm) selected from the apical to the basal turns of the cochlea were viewed under fluorescence microscopy and counted.

### Quantitative Real-Time PCR

TRIzol reagent was used to extract total RNA from HEI-OC-1 cells. The concentration and quality of RNA in the samples were measured by NanoDrop spectroscopy (Thermo NanoDrop 2000, United States). A BeyoRT^TM^ II First-Strand cDNA Synthesis Kit with gDNA Eraser was used for reverse transcription according to the instructions supplied by the manufacturer. SYBR Green was used to perform quantitative polymerase chain reaction (qPCR) on a real-time PCR instrument (cfx96, Bio-Rad). *Gapdh* was used as the internal reference gene. The primers were synthesized by Sangon Biotech (Shanghai, China). The primer sequences for the candidate genes are shown in Table 1.

**TABLE 1:** Primer sequences for qRT-PCR.

Gene	Sequence (5′ → 3′)	
*Gapdh*	F:	AGGTCGGTGTGAACGGATTTG
	R:	TGTAGACCATGTAGTTGPAGGTCA
*Caspase-3*	F:	ATGGAGAACAACAAAACCTCAGT
	R:	TTGCTCCCATGTATGGTCTTTAC
*Bax*	F:	TGAAGACAGGGGCCTTTTTG
	R:	AATTCGCCGGAGACACTCG
*Bcl-2*	F:	ATGCTTTGTGGAACTATATGGC
	R:	GGTATGCACCCAGAGTGATGC
*Alox15*	F:	GGCTCCAACAACGAGGTCTAC
	R:	AGGTATTCTGACACATCCACCTT
*Sod1*	F:	AACCAGTTGTGTTGTCAGGAC
	R:	CCACCATGTTTCTTAGAGTGAGG
*Caspase-9*	F:	CCTAGTGAGCGAGCTGCAAG
	R:	ACCGCTTTGCAAGAGTGAAG
*Gsr*	F:	TGCACTTCCCGGTAGGAAAC
	R:	GATCGCAACTGGGGTGAGAA
*Sirt1*	F:	TGTGGTGAAGATCTATGGAGGC
	R:	TGTACTTGCTGCAGACGTGGTA

*F, forward; R, reverse.*

### Immunofluorescence Assay

An antibody against cleaved CASPASE-3, the TUNEL Apoptosis Assay Kit, MitoSOX Red, the TMRE Mitochondrial Membrane Potential Assay Kit, an antibody against MYOSIN 7a (1:500 dilution), and DAPI (1:1,000 dilution) were used to analyze apoptotic cells, measure ROS, measure MMP, and stain HCs and nuclei. Briefly, after fixation with 4% paraformaldehyde at room temperature for 1 h, the cells and tissues were washed three times with PBST (1 × PBS containing 0.1% Triton X-100) and incubated in blocking medium (PBS containing 10% heat-inactivated donkey serum, 1% Triton X-100, 1% BSA, and 0.02% sodium azide, pH 7.2) at room temperature for 1 h. The samples were then incubated with primary antibodies against cleaved CASPASE-3 or MYOSIN 7a at 4°C for 8 h. After 3 washes with PBST, the samples were labeled with a secondary antibody for 1 h, washed again 3 times with PBST, mounted with a fluorescent blocking agent, and imaged with laser scanning confocal microscopy (Zeiss LSM700, Germany). TUNEL, MitoSox Red, and TMRE signals were measured according to the manufacturer’s instructions.

### Flow Cytometry Assay

We used DAPI and propidium iodide (PI) to distinguish dead cells from living cells. HEI-OC-1 cells were treated with neomycin in the presence or absence of GM1 and were then treated with trypsin and collected. The cells were washed twice, resuspended in PBS, and diluted to a concentration of 1 × 10^6^ cells/mL in PBS. DAPI and PI were added; the cells were then incubated in the dark at room temperature for 10–20 min and analyzed by flow cytometry (FACSCanto, BD) within 1 h. In addition, MitoSOX Red was used to determine the level of mitochondrial ROS.

### Statistical Analysis

All data are expressed as mean ± SD. All statistical analyses were performed using GraphPad Prism 7 software. Two-tailed and unpaired Student’s *t*-tests were used to assess the statistical significance of differences between the two groups. One-way ANOVA and Dunnett’s multiple comparison tests were used to compare multiple groups. *P* < 0.05 indicated a significant difference.

## Results

### Monosialotetrahexosylganglioside Protects HEI-OC-1 Cells From Neomycin-Induced Cytotoxicity

As shown in Figure 1, exposure to 2 mM neomycin decreased cell viability by approximately half in comparison with the control cells, and GM1 at 10–100 μM exhibited a certain degree of protection; 20 μM GM1 gave the strongest protection. At GM1 concentrations higher than 20 μM, the viability of HEI-OC-1 cells began to decrease (Figure 1B). To confirm this finding, we used a live-cell imaging system to observe HEI-OC-1 cells and found that the morphology of living cells in the 20 μM GM1 group was most similar to that of the cells in the control group (Figure 1C). Therefore, 20 μM GM1 was used as the treatment concentration in the follow-up experiments in this study.

### Monosialotetrahexosylganglioside Decreased Neomycin-Induced Apoptosis in HEI-OC-1 Cells

**FIGURE 2:**
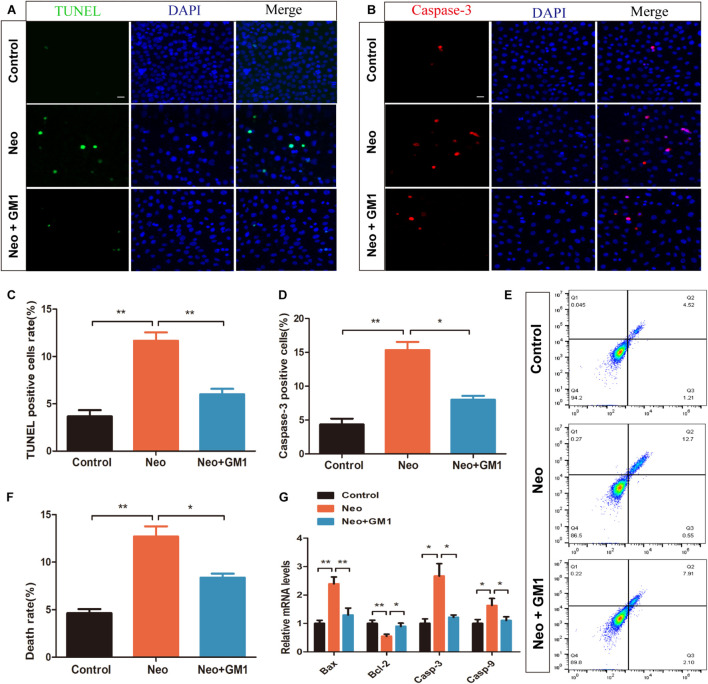
GM1 reduced neomycin-induced apoptosis in HEI-OC-1 cells. **(A)** Immunofluorescence images of apoptotic HEI-OC-1 cells with TUNEL and DAPI double staining. **(B)** Immunofluorescence images of apoptotic HEI-OC-1 cells with cleaved CASPASE-3 and DAPI double staining. **(C,D)** Quantification of the number of apoptotic cells in **(A,B)**. **(E,F)** Survival and percentage of HEI-OC-1 cells determined by DAPI/PI staining flow cytometry. **(G)** Expression of proapoptotic and antiapoptotic genes in HEI-OC-1 cells treated with neomycin and/or GM1 determined by qRT-PCR; the values were normalized to *Gapdh* as an internal control. The data are expressed as the mean ± SD of triplicate samples. *p < 0.05, **p < 0.01. Scale bars = 20 μm.

To confirm the protective effect of GM1 against apoptosis of HEI-OC-1 cells, HEI-OC-1 cells were stained using TUNEL and antibodies against cleaved CASPASE-3. The percentages of TUNEL-positive and cleaved CASPASE-3-positive cells in the neomycin group were significantly higher than those in the control group, and pretreatment with 20 μM GM1 significantly reduced the percentages of TUNEL-positive and cleaved CASPASE-3-positive cells after neomycin exposure (Figures 2A–D). Similarly, flow cytometry showed that 20 μM GM1 significantly reduced the death of HEI-OC-1 cells induced by neomycin (Figures 2E,F). The qRT-PCR results also showed that the mRNA expression of *Caspase-3, Bax*, and *Caspase-9* decreased significantly, while *Bcl-2* expression increased significantly, in the GM1 group compared with the neomycin group (Figure 2G). Taken together, our results suggest that GM1 protects HEI-OC-1 cells from neomycin-induced apoptosis.

### Monosialotetrahexosylganglioside Reduced Neomycin-Induced Apoptosis of Hair Cells in Explant-Cultured Cochleae

**FIGURE 3:**
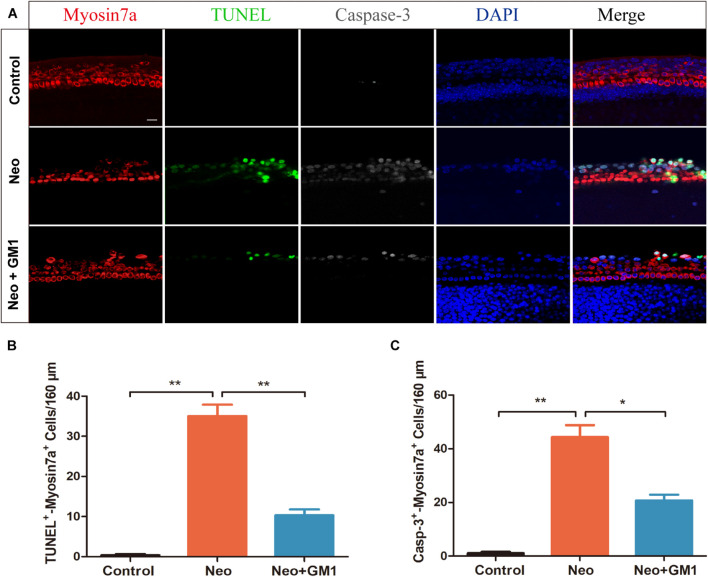
GM1 decreased neomycin-induced HC apoptosis in cochleae. **(A)** Immunofluorescence staining for TUNEL, cleaved CASPASE-3, and MYOSIN 7a in the basal turn of the cochlea in different treatment groups. **(B,C)** Quantification of the number of TUNEL/MYOSIN 7a and cleaved CASPASE-3/MYOSIN 7a double-positive cells per 160 μm of cochlea in **(A)**. The data are expressed as the mean ± SD of triplicate samples. **p < 0.01. Scale bars = 16 μm.

To explore the protective effect of GM1 on explant-cultured cochleae, TUNEL and antibody staining for cleaved CASPASE-3 were used to measure apoptosis of the cells in the explants after treatment. We found that the percentages of TUNEL-positive and cleaved CASPASE-3-positive cells in the neomycin group were significantly higher than those in the control group and that pretreatment with 20 μM GM1 significantly reduced the percentage of TUNEL-positive and cleaved CASPASE-3-positive cells after neomycin exposure (Figures 3A–C), consistent with the results obtained for HEI-OC-1 cells.

### Monosialotetrahexosylganglioside Protects Against Neomycin-Induced Hair Cell Loss in Explant-Cultured Cochleae

**FIGURE 4:**
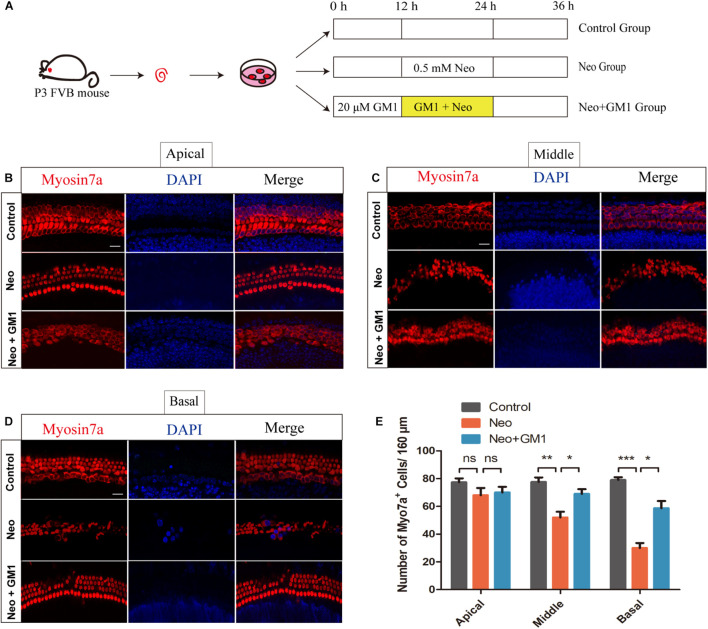
GM1 promoted the survival of cochlear HCs in explant cultures after neomycin exposure. **(A)** Schematic diagram illustrating GM1 and neomycin addition to explant-cultured cochleae. **(B,D)** Immunofluorescence staining of MYOSIN 7a/DAPI in the apical turns **(B)**, middle turns **(C)**, and basal turns **(D)** of explant-cultured cochleae in the different treatment groups. **(E)** Quantification of the number of MYOSIN 7a-positive cells per 160 μm of cochlea in **(B–D)**. The data are expressed as the mean ± SD of triplicate samples *p < 0.05, **p < 0.01, ***p < 0.001. Scale bars = 16 μm.

The procedures used to process the samples in the explant-cultured cochlea experiment are described in Figure 4A. MYOSIN 7a and DAPI staining were used to observe the changes in the number of HCs in the apical, middle and basal turns of the cochlea. We found that the number of HCs in the apical turn did not change significantly after neomycin exposure (Figure 4B). In contrast, the number of HCs in the middle and basal turns of the cochlea decreased significantly, and GM1 treatment successfully reduced HC loss in the middle and basal turns after neomycin exposure (Figures 4C–E).

### Monosialotetrahexosylganglioside Attenuates Oxidative Stress in Explant-Cultured Cochleae and HEI-OC-1 Cells After Neomycin Treatment

**FIGURE 5:**
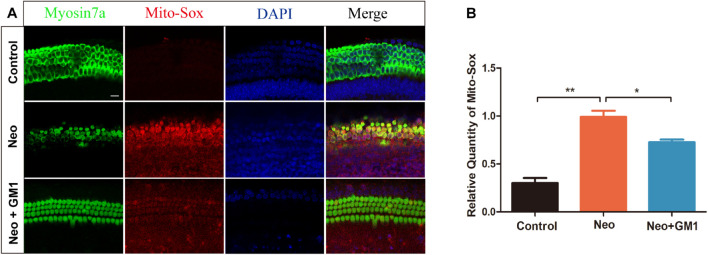
GM1 decreased ROS levels in explant-cultured cochlear HCs after neomycin exposure. **(A)** Immunofluorescence due to MitoSox, MYOSIN 7a and DAPI in the middle turns of explant-cultured cochleae in the different treatment groups. **(B)** Relative MitoSox immunofluorescence intensity. The data are expressed as the mean ± SD of triplicate samples. *p < 0.05, **p < 0.01. Scale bars = 16 μm.

**FIGURE 6:**
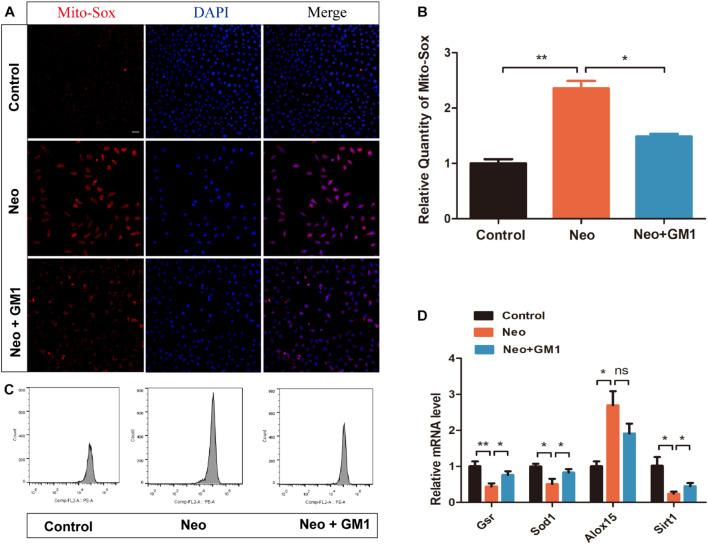
GM1 decreased ROS levels in HEI-OC-1 cells after neomycin exposure. **(A,B)** Immunofluorescence of MitoSox/DAPI in different treatment groups. **(C)** MitoSox levels of HEI-OC-1 cells in different treatment groups determined by flow cytometry. **(D)** mRNA expression levels of redox-related genes in HEI-OC-1 cells determined by qRT-PCR and normalized to *Gapdh*. The data are expressed as the mean ± SD of triplicate samples. *p < 0.05, **p < 0.01. Scale bars = 20 μm.

**FIGURE 7:**
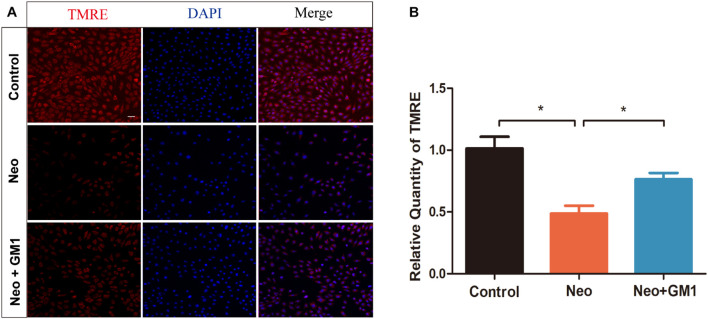
GM1 increases the mitochondrial membrane potential (MMP) after neomycin exposure. **(A)** Immunofluorescence of HEI-OC-1 cells in different treatment groups after labeling using a TMRE staining kit and DAPI. **(B)** Relative immunofluorescence intensities of the cells in **(A)**. The data are expressed as the mean ± SD of triplicate samples. *p < 0.05. Scale bars = 20 μm.

In this study, MitoSox Red immunofluorescence staining showed that the levels of ROS were significantly increased in the neomycin group compared with the control group and that pretreatment with 20 μM GM1 significantly reduced ROS levels compared with the neomycin group both in explant-cultured cochleae (Figures 5A,B) and in HEI-OC-1 cells (Figures 6A,B). MitoSOX flow cytometry of HEI-OC-1 cells yielded similar results (Figure 6C). qRT-PCR showed that the mRNA expression of *Gsr, Sod1*, and *Sirt1* was significantly upregulated in the GM1 pretreatment group compared with the neomycin group (Figure 6D). This suggests that GM1 alleviates the neomycin-induced imbalance in antioxidant-prooxidant gene expression. However, the mRNA expression of *Alox15* did not change significantly after GM pretreatment. We also found that the MMP decreased in the neomycin group but significantly increased in the GM1 group compared to that in the neomycin group, indicating that 20 μM GM1 treatment can prevent the MMP decrease induced by neomycin exposure in HEI-OC-1 cells (Figures 7A,B).

## Discussion

Gangliosides are mainly composed of hydrophobic ceramide and hydrophilic oligosaccharide chains containing sialic acid, which are fat-soluble and water-soluble, respectively. Ceramide is composed of long basic hydrophobic chains attached to fatty acids that are embedded in the lipid bilayer of the cell membrane and enhance the stability of the cell membrane structure. Oligosaccharides are linked to the serine residue of ceramide, and the sialic acid in oligosaccharides binds to peripheral calcium ions to maintain normal cellular physiological functions (Schnaar, 2016; Ledeen and Wu, 2018). Recent studies have reported that GM1 exerts antioxidant effects through its regulation of intracellular calcium levels in the hippocampus (She et al., 2009) and its activation of tyrosine kinase Trk receptors (Vlasova Iu et al., 2009) and that it has a protective effect on a variety of cells when applied as an intervention (Nikolaeva et al., 2015; Zhao et al., 2015). In this study, we used the HEI-OC-1 cell line and whole-organ explant cultures to investigate the protective effect of GM1 on neomycin-induced HC injury *in vitro*. Our results showed that a protective effect of GM1 on HEI-OC-1 cells is observed when GM1 is applied at concentrations between 10 and 100 μM and that 20 μM is the optimal concentration; beyond this, the protective effect begins to decline, suggesting that GM1 can protect HCs when applied at an appropriate concentration.

Previous studies have suggested that aminoglycoside drugs induce HEI-OC-1 cell apoptosis by promoting the release of proapoptotic factors (Leis et al., 2015). Our previous research also showed that after exposure of HEI-OC-1 cells to neomycin, the expression of proapoptotic genes such as *Caspase 3* and *Bax* increased significantly, while that of the antiapoptotic genes *Bcl-2* and *Nf-kb* decreased significantly (Gao et al., 2019). In the current study, we found that treatment with GM1 decreases the death and apoptosis induced by neomycin. Moreover, our results also showed that exposure of cochlear explant cultures to GM1 protected HC cells in the middle and basal turns of the cochlea from damage; since the damage caused by aminoglycoside drugs shows a decreasing trend from the basal turn to the apical turn of the cochlea (Zhang et al., 2019) and high-frequency hearing loss is the first and most severe impairment in patients with ototoxic hearing loss (Guo et al., 2019), the protective effect of GM1 on high-frequency hearing may have potential clinical significance.

BCL-2 family proteins play a key role in the apoptosis process. Proapoptotic proteins such as BAX change their molecular conformations when they encounter apoptosis signals; they then translocate and insert into the outer mitochondrial membrane, eventually leading to increased permeability of the outer mitochondrial membrane, the release of cytochrome c (Cyt C), and the activation of multiple caspases. BCL-2 can prevent this process (Verma and Syed Mohammed, 2019; Tian et al., 2020; Zeng et al., 2020). Our study found that the mRNA expression of *Bcl-2* was upregulated and that that of *Bax* was downregulated, indicating that GM1 can relieve neomycin-induced HC damage by regulating the mRNA expression of *Bax* and *Bcl-2*. This is consistent with the report by Chen et al. (2019) that GM1 increases *Bcl-2* expression and reduces *Bax* expression, thereby protecting against lead-induced neurological damage in developing rats.

Many studies have found that aminoglycoside antibiotics aggregate in inner ear HCs, where they affect normal metabolism and produce a large number of highly toxic ROS (Han et al., 2020). ROS include hydroxyl radicals (OH∙), hydrogen peroxide, superoxide and other free radicals, all of which exhibit great oxygen toxicity. ROS can affect various signaling pathways and physiological activities of cells, including cell growth, division, differentiation and apoptosis. Excessive ROS levels can lead to a variety of diseases (Zhang et al., 2016). Studies have found that aminoglycosides bind to calreticulin, which is highly expressed in cochlear marginal cells and HCs. and disrupt chaperone activity. This binding in turn elevates mitochondrial Ca^2+^ levels, and this leads to elevated levels of both mitochondrial oxidation and cytoplasmic ROS and to an imbalance in the redox state (Jiang et al., 2017). The high reactivity of oxygen free radicals can damage cell components and structures such as nucleic acids and cell membranes. ROS accumulation can also cause mitochondrial depolarization and change mitochondrial membrane permeability. The resulting impairment of mitochondrial function leads to the further accumulation of oxygen free radicals and eventually to apoptosis (He et al., 2020; Zhong et al., 2020). In this study, we found that GM1 significantly decreased ROS levels in HEI-OC-1 cells and explant-cultured cochlear HCs, suggesting that GM1 can alleviate mitochondrial dysfunction after exposure to neomycin. Redox balance plays an important role in the generation and elimination of ROS. In this study, the mRNA expression levels of *Gsr, Sod1*, and *Sirt1* decreased and that of *Alox15* increased significantly after neomycin exposure, and GM1 pretreatment significantly improved the imbalance in the mRNA expression of *Gsr, Sod1*, and *Sirt1* induced by neomycin. These results suggest that exposure of cells to GM1 can correct the imbalance in the expression of prooxidant and antioxidant genes observed after neomycin exposure, thereby reducing the ROS level and preventing HC mitochondrial dysfunction and apoptosis. However, it must be noted that the use of high GM1 concentrations will reduce the protective effect and may cause additional damage to HCs. Drug dosage must be considered in future animal and clinical studies.

In conclusion, our results suggest that GM1 can maintain the normal balance between oxidative and antioxidant gene expression, reduce the accumulation of ROS, and reduce apoptosis after exposure of HEI-OC-1 cells and cultured cochlear HCs to neomycin. These results indicate that GM1 may have potential clinical value in protecting against hearing impairment in individuals treated with aminoglycoside drugs.

## Data Availability Statement

The original contributions presented in the study are included in the article/supplementary material, further inquiries can be directed to the corresponding author/s.

## Ethics Statement

The animal study was reviewed and approved by the Animal Care and Use Committee of Nanjing Medical University.

## Author Contributions

YL, AL, and CW performed the experiments, analyzed the data, and wrote the manuscript. LL, S-LW, and XG designed the experiments and provided critical revision. XJ and YZ reviewed the experimental data and conducted the statistical analysis. All authors contributed to the article and approved the submitted version.

## Conflict of Interest

The authors declare that the research was conducted in the absence of any commercial or financial relationships that could be construed as a potential conflict of interest.

## Publisher’s Note

All claims expressed in this article are solely those of the authors and do not necessarily represent those of their affiliated organizations, or those of the publisher, the editors and the reviewers. Any product that may be evaluated in this article, or claim that may be made by its manufacturer, is not guaranteed or endorsed by the publisher.

## References

[B1] Chen C. Wang S. Liu P. (2019). Deferoxamine enhanced mitochondrial iron accumulation and promoted cell migration in triple-negative MDA-MB-231 breast cancer cells via a ROS-dependent mechanism. *Int. J. Mol. Sci.* 20:4952. 10.3390/ijms20194952 31597263 PMC6801410

[B2] Diao Y. Dong M. (2012). The observation on curative effect of 90 cases of sudden hearing loss. *Lin chuang er bi yan hou tou jing wai ke za zhi* 36 306–308. 22737871

[B3] Fazzari M. Audano M. Lunghi G. Di Biase E. Loberto N. Mauri L. (2020). The oligosaccharide portion of ganglioside GM1 regulates mitochondrial function in neuroblastoma cells. *Glycoconj J.* 37 293–306. 10.1007/s10719-020-09920-4 32266604

[B4] Franco D. A. Truran S. Weissig V. Guzman-Villanueva D. Karamanova N. Senapati S. (2016). Monosialoganglioside-containing nanoliposomes restore endothelial function impaired by AL amyloidosis light chain proteins. *J. Am. Heart Assoc.* 5:e003318. 10.1161/JAHA.116.003318 27412900 PMC4937272

[B5] Gao S. Cheng C. Wang M. Jiang P. Zhang L. Wang Y. (2019). Blebbistatin inhibits neomycin-induced apoptosis in hair cell-like HEI-OC-1 cells and in cochlear hair cells. *Front. Cell. Neurosci.* 13:590. 10.3389/fncel.2019.00590 32116554 PMC7025583

[B6] Germovsek E. Barker C. I. Sharland M. (2017). What do I need to know about aminoglycoside antibiotics? *Arch. Dis. Child Educ. Pract. Ed.* 102 89–93. 10.1136/archdischild-2015-309069 27506599

[B7] Guo J. Chai R. Li H. Sun S. (2019). Protection of hair cells from ototoxic drug-induced hearing loss. *Adv. Exp. Med. Biol.* 1130 17–36. 10.1007/978-981-13-6123-4_2 30915699

[B8] Guo Y. L. Duan W. J. Lu D. H. Ma X. H. Li X. X. Li Z. (2021). Autophagy-dependent removal of α-synuclein: a novel mechanism of GM1 ganglioside neuroprotection against Parkinson’s disease. *Acta Pharmacol. Sin.* 42 518–528. 10.1038/s41401-020-0454-y 32724177 PMC8115090

[B9] Han H. Dong Y. Ma X. (2020). Dihydromyricetin protects against gentamicin-induced ototoxicity via PGC-1α/SIRT3 Signaling in vitro. *Front. Cell. Dev. Biol.* 8:702. 10.3389/fcell.2020.00702 32850822 PMC7399350

[B10] He Y. Li W. Zheng Z. Zhao L. Li W. Wang Y. (2020). Inhibition of Protein arginine methyltransferase 6 reduces reactive oxygen species production and attenuates aminoglycoside- and cisplatin-induced hair cell death. *Theranostics* 10 133–150. 10.7150/thno.37362 31903111 PMC6929624

[B11] Jiang M. Karasawa T. Steyger P. S. (2017). Aminoglycoside-Induced cochleotoxicity: A review. *Front. Cell Neurosci.* 11:308. 10.3389/fncel.2017.00308 29062271 PMC5640705

[B12] Jiang P. Ray A. Rybak L. P. Brenner M. J. (2016). Role of STAT1 and oxidative stress in gentamicin-induced hair cell death in organ of corti. *Otol. Neurotol.* 37 1449–1456. 10.1097/MAO.0000000000001192 27631653 PMC5125081

[B13] Ledeen R. Wu G. (2018). Gangliosides of the nervous system. *Methods Mol. Biol.* 1804 19–55. 10.1007/978-1-4939-8552-4_2 29926403

[B14] Leis J. A. Rutka J. A. Gold W. L. (2015). Aminoglycoside-induced ototoxicity. *Curr. Pharm. Des.* 187:E52. 10.1503/cmaj.140339 25225217 PMC4284193

[B15] Li A. You D. Li W. Cui Y. He Y. Li W. (2018). Novel compounds protect auditory hair cells against gentamycin-induced apoptosis by maintaining the expression level of H3K4me2. *Drug Deliv.* 25 1033–1043. 10.1080/10717544.2018.1461277 30799660 PMC6058728

[B16] Lopez-Novoa J. M. Quiros Y. Vicente L. Morales A. I. Lopez-Hernandez F. J. (2011). New insights into the mechanism of aminoglycoside nephrotoxicity: an integrative point of view. *Kidney Int.* 79 33–45. 10.1038/ki.2010.337 20861826

[B17] Magistretti P. J. Geisler F. H. Schneider J. S. Li P. A. Fiumelli H. Sipione S. (2019). Gangliosides: treatment avenues in neurodegenerative disease. *Front. Neurol.* 10:859. 10.3389/fneur.2019.00859 31447771 PMC6691137

[B18] Nikolaeva S. Bayunova L. Sokolova T. Vlasova Y. Bachteeva V. Avrova N. (2015). GM1 and GD1a gangliosides modulate toxic and inflammatory effects of E. coli lipopolysaccharide by preventing TLR4 translocation into lipid rafts. *Biochim. Biophys. Acta* 1851 239–247. 25499607 10.1016/j.bbalip.2014.12.004

[B19] Pham T. N. M. Jeong S. Y. Kim D. H. Park Y. H. Lee J. S. Lee K. W. (2020). Protective mechanisms of avocado oil extract against ototoxicity. *Nutrients* 12:947. 10.3390/nu12040947 PMC7230542 32235401

[B20] Rao S. Lin Y. Du Y. He L. Huang G. Chen B. (2019). Designing multifunctionalized selenium nanoparticles to reverse oxidative stress-induced spinal cord injury by attenuating ROS overproduction and mitochondria dysfunction. *J. Mater Chem. B* 7 2648–2656. 10.1039/c8tb02520g 32254998

[B21] Schnaar R. L. (2016). Gangliosides of the vertebrate nervous system. *J. Mol. Biol.* 428 3325–3336. 10.1016/j.jmb.2016.05.020 27261254 PMC4983208

[B22] She J. Q. Wang M. Zhu D. M. Tang M. Chen J. T. Wang L. (2009). Monosialoanglioside (GM1) prevents lead-induced neurotoxicity on long-term potentiation, SOD activity, MDA levels, and intracellular calcium levels of hippocampus in rats. *Naunyn Schmiedebergs Arch. Pharmacol.* 379 517–524. 10.1007/s00210-008-0379-3 19043692

[B23] Sotoudeh H. (2021). Hearing loss prevalence and years lived with disability, 1990–2019: findings from the Global Burden of Disease Study 2019. *Lancet* 397 996–1009. 33714390 10.1016/S0140-6736(21)00516-X PMC7960691

[B24] Tanaka S. Tabuchi K. Hoshino T. Murashita H. Tsuji S. Hara A. (2010). Protective effects of exogenous GM-1 ganglioside on acoustic injury of the mouse cochlea. *Neurosci. Lett.* 473 237–241. 10.1016/j.neulet.2010.02.057 20219629

[B25] Tian J. Mo J. Xu L. Zhang R. Qiao Y. Liu B. (2020). Scoulerine promotes cell viability reduction and apoptosis by activating ROS-dependent endoplasmic reticulum stress in colorectal cancer cells. *Chem. Biol. Interact* 327:109184. 10.1016/j.cbi.2020.109184 32590070

[B26] Verma J. Syed Mohammed T. (2019). Evaluating hearing loss in patients undergoing second line anti tubercular treatment. *Indian J. Otolaryngol. Head Neck Surg.* 71 1202–1206. 10.1007/s12070-018-1266-y 31750151 PMC6841865

[B27] Vlasova Iu A. Zakharova I. O. Sokolova T. V. Furaev V. V. Rychkova M. P. Avrova N. F. (2009). Role of tyrosine kinase of Trk-receptors in realization of antioxidant effect of ganglioside GM1 in PC12 cells. *Zh. Evol. Biokhim. Fiziol.* 45 465–471. 19886192

[B28] Yao L. Zhang J. W. Chen B. Cai M. M. Feng D. Wang Q. Z. (2020). Mechanisms and pharmacokinetic/pharmacodynamic profiles underlying the low nephrotoxicity and ototoxicity of etimicin. *Acta Pharmacol. Sin.* 41 866–878. 31937930 10.1038/s41401-019-0342-5 PMC7468263

[B29] Zeng H. Kong X. Zhang H. Chen Y. Cai S. Luo H. (2020). Inhibiting DNA methylation alleviates cigarette smoke extract-induced dysregulation of Bcl-2 and endothelial apoptosis. *Tob. Induc. Dis.* 18:51. 10.18332/tid/119163 32547354 PMC7291961

[B30] Zhang J. Wang X. Vikash V. Ye Q. Wu D. Liu Y. (2016). ROS and ROS-mediated cellular signaling. *Oxid Med. Cell Longev.* 2016:4350965. 10.1155/2016/4350965 26998193 PMC4779832

[B31] Zhang S. Qiang R. Dong Y. Zhang Y. Chen Y. Zhou H. (2020). Hair cell regeneration from inner ear progenitors in the mammalian cochlea. *Am. J. Stem Cells* 9 25–35. 32699655 PMC7364385

[B32] Zhang Y. Li W. He Z. Wang Y. Shao B. Cheng C. (2019). Pre-treatment with fasudil prevents neomycin-induced hair cell damage by reducing the accumulation of reactive oxygen species. *Front. Mol. Neurosci.* 12:264. 10.3389/fnmol.2019.00264 31780893 PMC6851027

[B33] Zhao H. Li X. Li G. Sun B. O. Ren L. Zhao C. (2015). Protective effects of monosialotetrahexosylganglioside sodium on H2O2-induced human vascular endothelial cells. *Exp. Ther. Med.* 10 947–953. 10.3892/etm.2015.2603 26622420 PMC4533136

[B34] Zhong Z. Fu X. Li H. Chen J. Wang M. Gao S. (2020). Citicoline protects auditory hair cells against neomycin-induced damage. *Front. Cell. Dev. Biol.* 8:712. 10.3389/fcell.2020.00712 32984303 PMC7487320

